# Comparative metabolomics studies of blood collected in streck and heparin tubes from lung cancer patients

**DOI:** 10.1371/journal.pone.0249648

**Published:** 2021-04-23

**Authors:** Erin Goldberg, Shiva Ievari-Shariati, Biniam Kidane, Julian Kim, Shantanu Banerji, Gefei Qing, Sadeesh Srinathan, Leigh Murphy, Michel Aliani

**Affiliations:** 1 Department of Food and Human Nutritional Sciences, University of Manitoba, Winnipeg, MB, Canada; 2 The Canadian Centre for Agri-Food Research in Health and Medicine, (CCARM), St. Boniface Hospital Albrechtsen Research Centre, Winnipeg, MB, Canada; 3 Department of Physiology and Pathophysiology, University of Manitoba, Winnipeg, MB, Canada; 4 Department of Surgery, Rady Faculty of Health Sciences, University of Manitoba, Winnipeg, MB, Canada; 5 Department of Radiology, CancerCare Manitoba, Winnipeg, MB, Canada; 6 Research Institute in Oncology and Hematology, CancerCare Manitoba, Winnipeg, MB, Canada; 7 Department of Pathology, Rady Faculty of Health Sciences, University of Manitoba, Winnipeg, MB, Canada; 8 Research Institute in Oncology and Hematology, CancerCare Manitoba, Department of Biochemistry & Medical Genetics, Rady Faculty of Health Sciences, University of Manitoba, Winnipeg, MB, Canada; Weill Cornell Medical College QATAR

## Abstract

Metabolomics analysis of blood from patients (n = 42) undergoing surgery for suspected lung cancer was performed in this study. Venous and arterial blood was collected in both Streck and Heparin tubes. A total of 96 metabolites were detected, affected by sex (n = 56), collection tube (n = 33), and blood location (n = 8). These metabolites belonged to a wide array of compound classes including lipids, acids, pharmaceutical agents, signalling molecules, vitamins, among others. Phospholipids and carboxylic acids accounted for 28% of all detected compounds. Out of the 33 compounds significantly affected by collection tube, 18 compounds were higher in the Streck tubes, including allantoin and ketoleucine, and 15 were higher in the Heparin tubes, including LysoPC(P-16:0), PS 40:6, and chenodeoxycholic acid glycine conjugate. Based on our results, it is recommended that replicate blood samples from each patient should be collected in different types of blood collection tubes for a broader range of the metabolome. Several metabolites were found at higher concentrations in cancer patients such as lactic acid in Squamous Cell Carcinoma, and lysoPCs in Adenocarcinoma and Acinar Cell Carcinoma, which may be used to detect early onset and/or to monitor the progress of the cancer patients.

## Introduction

Lung cancer is the most commonly diagnosed cancer in Canada, excluding non-melanoma skin cancers [1]. It is the leading cause of cancer-related death for both men (25.2% of cancer deaths) and women (26.1% of cancer deaths) in Canada. There are two types of lung cancer: small cell lung cancer (SCLC) and non-small cell lung cancer (NSCLC), with the latter accounting for ~90% of all LC diagnoses and typically grow at a much slower rate than the SCLC. NSCLC accounts for ~85% of all LC diagnoses. The main subtypes of NSCLC include adenocarcinoma (AC) (40% of LC), squamous cell carcinoma (SCC) (25% of LC) and large cell carcinoma (10% of LC). Acinar cell adenocarcinoma (ACA) is a type of LC of the salivary type and extremely rare. There are also numerous, additional rare subtypes of decreasing frequency, as defined by the World Health Organization [2].

Most clinical and research samples in lung cancer are collected using venous blood collected through standard venipuncture techniques used in routine clinical care. Arterial blood is rarely collected due to greater patient discomfort and technical challenges, restricting collection to higher acuity settings such as the operating room. Serial venous samples often require repeated venipuncture for collection. Arterial catheters are routinely placed during lung cancer surgery; thus, arterial sampling may represent an opportunity to achieve single or serial sampling without need for patients to endure multiple punctures.

Standard clinical peripheral blood collection uses sodium Heparin (a glycosaminoglycan, polysaccharide) as the anticoagulant. Heparin blood collection tubes (BCT) are widely used for metabolomic analysis. Streck BCT have emerged as the current ‘gold standard’ for collecting peripheral blood for analysis of cfDNA and CTCs. Streck BCT use an EDTA-based anticoagulants and also contain a proprietary non-formaldehyde based cocktail that stabilizes nucleated white blood cells [3]. This keeps the plasma clean from genomic DNA caused by cellular degradation thereby maximizing the detection of cfDNA is relevant for cancer diagnostics. These tubes provide a cleaner extract, with potential application to metabolomic analysis. However, no studies to date have shown that Streck BCT are suitable for this type of analysis.

The current study was undertaken to determine changes in the metabolomic profiles of venous blood and arterial blood.

The objectives in this study were two-fold, to investigate:

1. The effect of blood collection tube (Streck *vs*. Heparin), blood location (venous *vs*. arterial), and sex, on metabolic profile of the 42 cancer patients.
2. The metabolic profile of cancer patients suffering from AC, SCC and ACA compared to benign tumours (a pilot study).

## Materials and methods

We conducted high-performance liquid chromatography/quadruple time-of-flight mass spectrometry (HPLC-QTOF-MS) analysis on a cohort of 42 patients undergoing surgery for suspected lung cancer. These patients consented to provide concurrent arterial and venous blood at the time of surgery, each collected in Streck and Heparin tubes.

### Chemicals

Citric acid-*d*_4_, L-leucine-*d*_10_, D-fructose -^13^C_6_, L-tryptophan-*d*_5_, L-valine-*d*_3_, glycerol-*d*_3_, benzoic acid-*d*_5_, stearic acid-*d*_35_, Norvaline, HPLC grade acetone and HPLC grade methanol were all purchased from Sigma Aldrich (St. Louis, MO). L-Alanine-*d*_4_ was purchased from Isotec (Miamisburg, OH). Optima grade acetonitrile was purchased from Fisher Scientific (Fair Lawn, NJ).

Two solutions were prepared to serve as internal standards using all deuterated compounds. The first solution, deuterated standard solution, contained all compounds except for stearic acid-*d*_35_ at a concentration of 15 ng/μL dissolved in 50:50 MeOH:dH_2_O. The second solution contained stearic acid-*d*_35_ at a concentration of 60 ng/μL dissolved in acetone. A reconstitution solution was prepared as 4:1 acetonitrile:H_2_O (milliQ) with norvaline added at a concentration of 1.5 ng/μL.

### Blood collection

The Manitoba Tumour Bank (MTB) operates with approval from the Health Research Ethics Board of the University of Manitoba (HS14357 (H2006:152)) and the MTB is a founding member of and certified by the Canadian Tissue Repository Network (www.CTRNet.ca). All patients provided informed written consent to have their samples and associated health information used in research. The MTB CancerCare Manitoba provided blood samples and health information from 42 patients. The study was conducted with the approval of the Health Research Ethics Board of the University of Manitoba (HS21714(H2018:151)). The patient data were accessed between Oct 2017 and Aug 2020, from CancerCare Manitoba health databases. All samples and associated data were stripped of all personal identifiers and released to the study using a number assigned by the MTB.

Blood was collected from patients at the time of surgery for suspected lung cancer. The characteristics of these patients are summarized in Table 1.

**Table 1:** Patient characteristics (N = 42).

**Female: Male**	50%-50%
**Median Age of Diagnosis**	70 (25–83) years
**Histology**	
Adenocarcinoma	52.4% (22)
• Adenocarcinoma	16.7% (7)
• Acinar Cell Adenocarcinoma	16.7% (7)
• Other	19.1% (8)
Squamous Cell Carcinoma	26.2% (11)
Other–Carcinoid	4.8% (2)
Benign	7.1% (3)
Metastasis from other primary	9.5% (4)
Stage (includes carcinoid tumours but does not include metastasis from other primary)	
**1a**	48.6% (17)
**1b**	17.1% (6)
**IIa**	8.6% (3)
**IIb**	5.7% (2)
**III**	11.4% (4)
**IV**	8.6% (3)
**Smoking Status**	
Never	14.3% (6)
Current	21.4% (9)
Ever	64.3% (27)

Arterial blood was drawn from the indwelling radial arterial line into 10 ml Streck BCT (STRECK LaVista NE, USA) or 10 ml sodium Heparin Vacutainer BCT (Becton and Dickinson Franklin Lakes, NJ, USA). Venous blood was taken from the forearm (median cubital, cephalic, or basilica veins) at the same time into a 10 ml Streck tube or a 10 ml sodium Heparin Vacutainer tube. The tubes were inverted 3–4 times and delivered promptly to the MTB for processing. Samples were anonymized using a unique MTB number. The tubes were centrifuged at 450 x g for 10 minutes at room temperature. The plasma was carefully removed and dispensed into 0.5 ml aliquots in Simport cryovials, frozen and stored at -80°C until further analysis. All blood collected for the study was processed and frozen within 2 hours of collection.

### Extraction procedure

Frozen samples (~0.5mL) were removed from storage at -80°C and placed on ice. Six samples were processed in one batch. While thawing, 20μL of the deuterated standard solution and 5μL of the stearic acid solution were added to 1.5 mL microcentrifuge tubes (Basix–Fisher Scientific, Fair Lawn, NJ).

The 200μL was split into two duplicates so that 100μL was added to two different tubes. Ice cold acetonitrile (200μL) was added to each tube, and then each tube was vortexed at 2500 rpm for 30 seconds on a LP Vortex Mixer (Fisher Scientific, Fair Lawn, NJ). Immediately following vortexing, the samples were loaded into a Benchmark Z216 MK Refrigerated Microcentrifuge (Hermle, Gosheim, Germany) and spun at 10,000g for 10 minutes at 4°C. When complete, the supernatant was transferred to a new microcentrifuge tube leaving behind a small drop of immiscible liquid and condensed pellet. The supernatant was dried under a gentle stream of nitrogen from a nitrogen evaporator (Lab-Line, Dubuque, IA). Dried extracts were stored at -20°C until ready to be run on the LC-QTOF/MS. When samples were ready to be run, 100μL of reconstitution solution was added and then samples were vortexed for 4 minutes at 2500 rpm. The samples were centrifuged once more at 10,000g for 5 minutes, and transferred to 250μL glass inserts and kept in 2mL amber vials.

### Instrumental method

Following extraction, samples were analyzed on a Rapid Resolution HPLC system (1290 Infinity Agilent Ltd., Santa Clara, Ca) equipped with a binary pump, degasser, well-plate auto-sampler (maintained at 6°C throughout the runs) with a thermostat and thermostatic column. This system was coupled to a 6538 UHD Accurate LC-QTOF-MS (Agilent Technologies, Santa Clara CA) with dual electrospray ionization (ESI) source. A 2.1 mm x 50 mm Agilent ZORBAX Extend C-18 column (Agilent Technologies, Santa Clara, CA) was maintained at 55°C for chromatographic separation of the plasma extracts. Two mobile phases, water (A) and acetonitrile (B) were used and both contained 0.1% formic acid. The solvent program started at 70% A and held for half a minute. Then A was decreased to 0% up until 12 minutes and held for another half minute. The percentage of A was then increased back up to 70% over 30 sec. for a total run time of 13 minutes. A post-run time of 1 minute was added to re-equilibrate the column between runs. Each sample had a 2 μL injection and the flow rate was held at 0.4 mL/min.

MS data acquisitions were completed in both positive (+) and negative (-) ESI modes. MS parameters included capillary voltage (4000 V), the fragmentor (175V), the skimmer (50V), and the OCT 1 RFVpp (750 V). For drying, nitrogen gas (N2) was utilized at 11 L/min at 300°C with nebulizer settings at 50 psig. MS spectra were collected within the range of 50–1700 m/z and known reference masses of 121.0508 and 922.0097 (ESI+) and 112.9860 and 1033.9880 (ESI-) were utilized during all runs.

### Data processing

The LC/MS data files were processed by Agilent Mass Hunter Qualitative (MHQ, Version B.07) to generate total ion current (TIC) chromatograms and the raw data stored as “.d” formats. The chromatograms were searched for ~2800 endogenous compounds by their formulas. The results from the searches were summarized in and converted to “.cef” files. Mass Professional Profiler (MPP, Version 12.6) was used for further data processing. The peaks were aligned to correct for slight variations in retention time (0.5 min) and normalized using a percentile shift algorithm (75.0) and were baselined using the baseline to median (means across samples) option. Metlin Database was used for the identification of the metabolites. A 10 ppm database mass accuracy score was used to annotate all metabolites using accurate mass and isotope ratios.

### Statistical analysis

Three-way ANOVA (p<0.05) was performed using location of the blood sampling (n = 2), the collection tubes (n = 2), and sex (n = 2) as the three tested factors. One-way ANOVA (p<0.05) was performed to examine the metabolic profile of cancer patients suffering from AC, SCC and ACA compared to benign tumours. Bonferroni FWER multiple tests correction was applied in both cases. Mass Professional Profiler (MPP, Version 12.6) was used for all statistical analyses including the partial least square discrimination (PLSD) analysis and matrix plots analysis. XLSTAT (Premium, Version 19.4) was used to generate hierarchical clustering heatmaps and principal component analysis (PCA) biplots. A summary of the metabolomics workflow used for objectives 1 and 2 is provided in Table 2.

**Table 2:** The metabolomics workflow applied to blood samples collected from 42 cancer patients.

**Fig 1:**
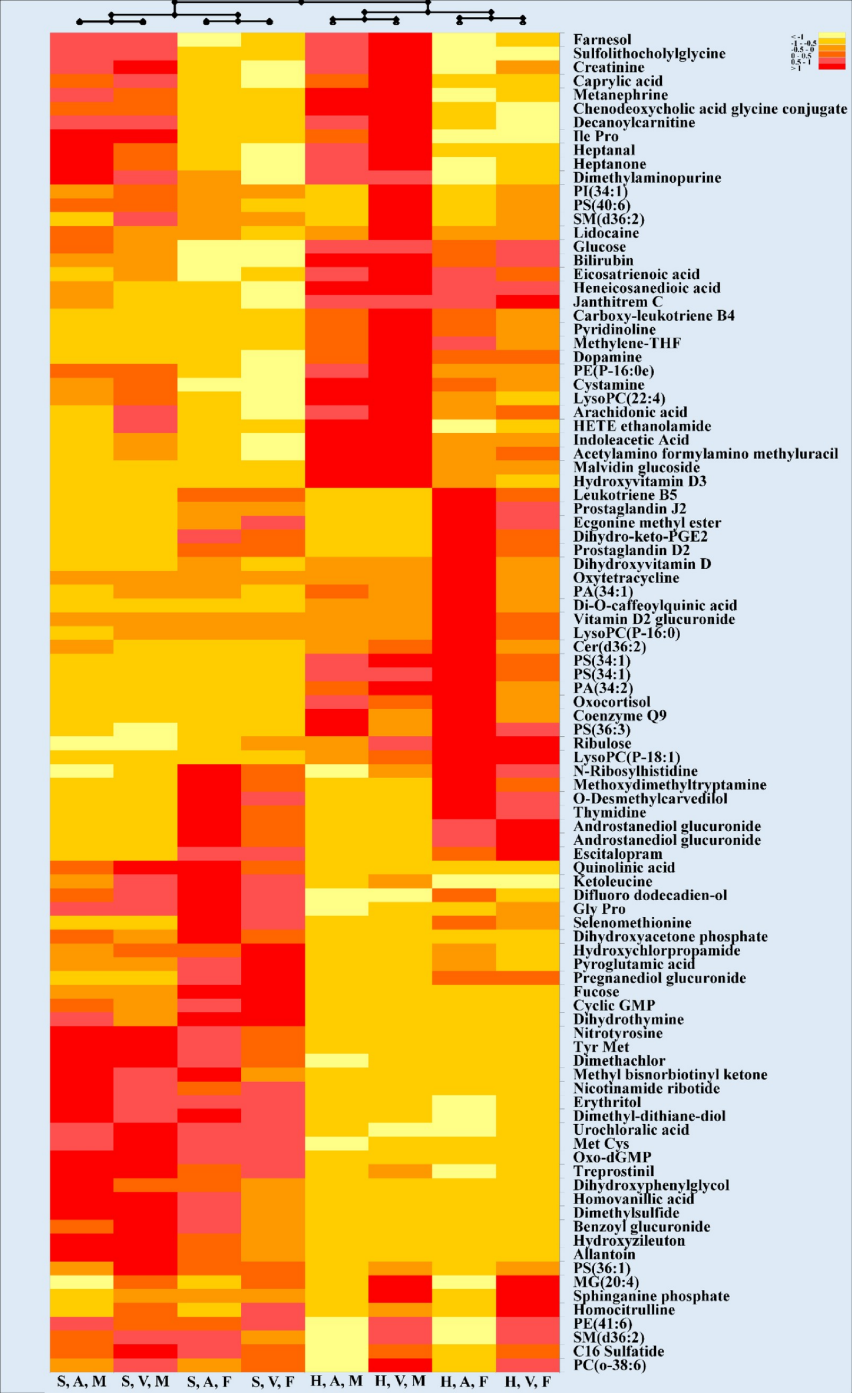
Hierarchical clustering heatmap representing the effect of blood collection tube, blood location and sex in 42 cancer patients. Each row represents a metabolite (total of 96 significant metabolites after statistical analysis; three-way ANOVA; p< 0.05; Bonferroni FWER). Values for each cell represents Log 2-normalized values for each metabolite. Heatmap criterion: Interquartile range < /Threshold = 0.25. Abbreviations: S: Streck; H: Heparin; A: Arterial; V: Venous; M: Male, F: Female.

**Fig 2:**
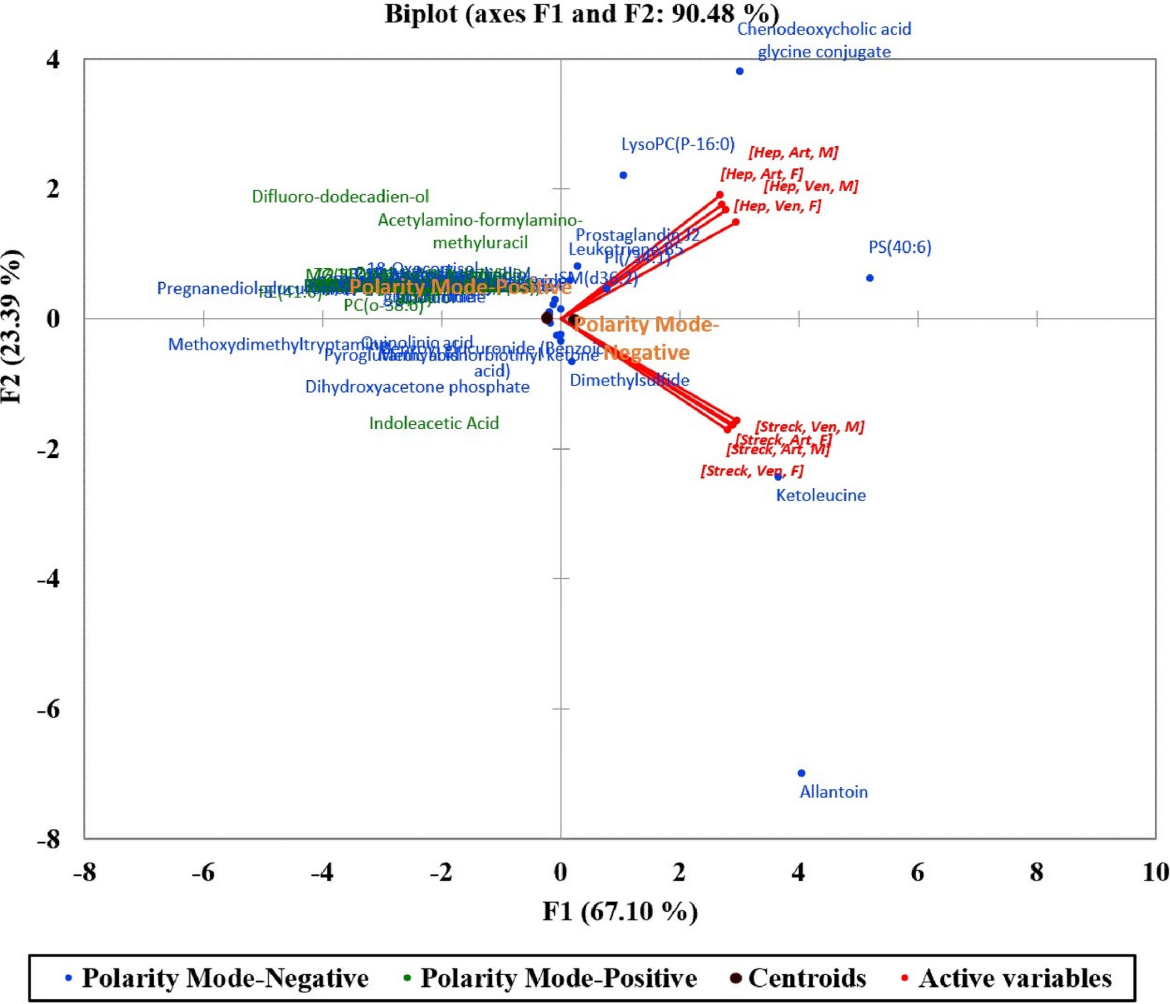
Principal component analysis (PCA) biplot (axes F1 and F2: 90.48%) of detected metabolites collected from 42 patients.

**Fig 3:**
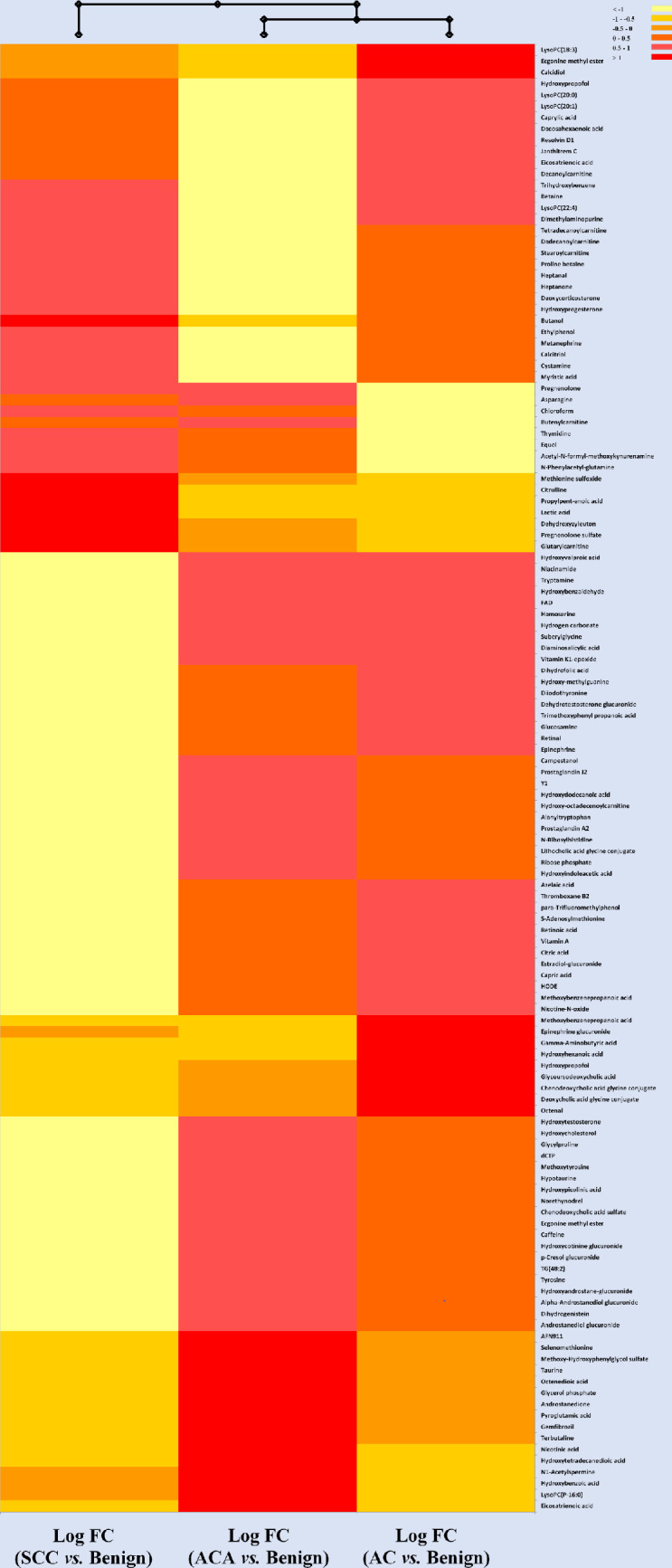
Hierarchical clustering heatmap representing the metabolic profile of cancer patients with SCC, ACA and AC versus patients with benign tumours. Each row represents a metabolite (total of 96 significant metabolites after statistical analysis; One-Way ANOVA; p< 0.05; Bonferroni FWER). Values for each cell represents Log 2 Fold Change (FC)-normalized values for the ratio of each of the three cancer types (SCC, ACA or AC)/ Benign. Heatmap criterion: Interquartile range </Threshold = 0.25.

**Fig 4:**
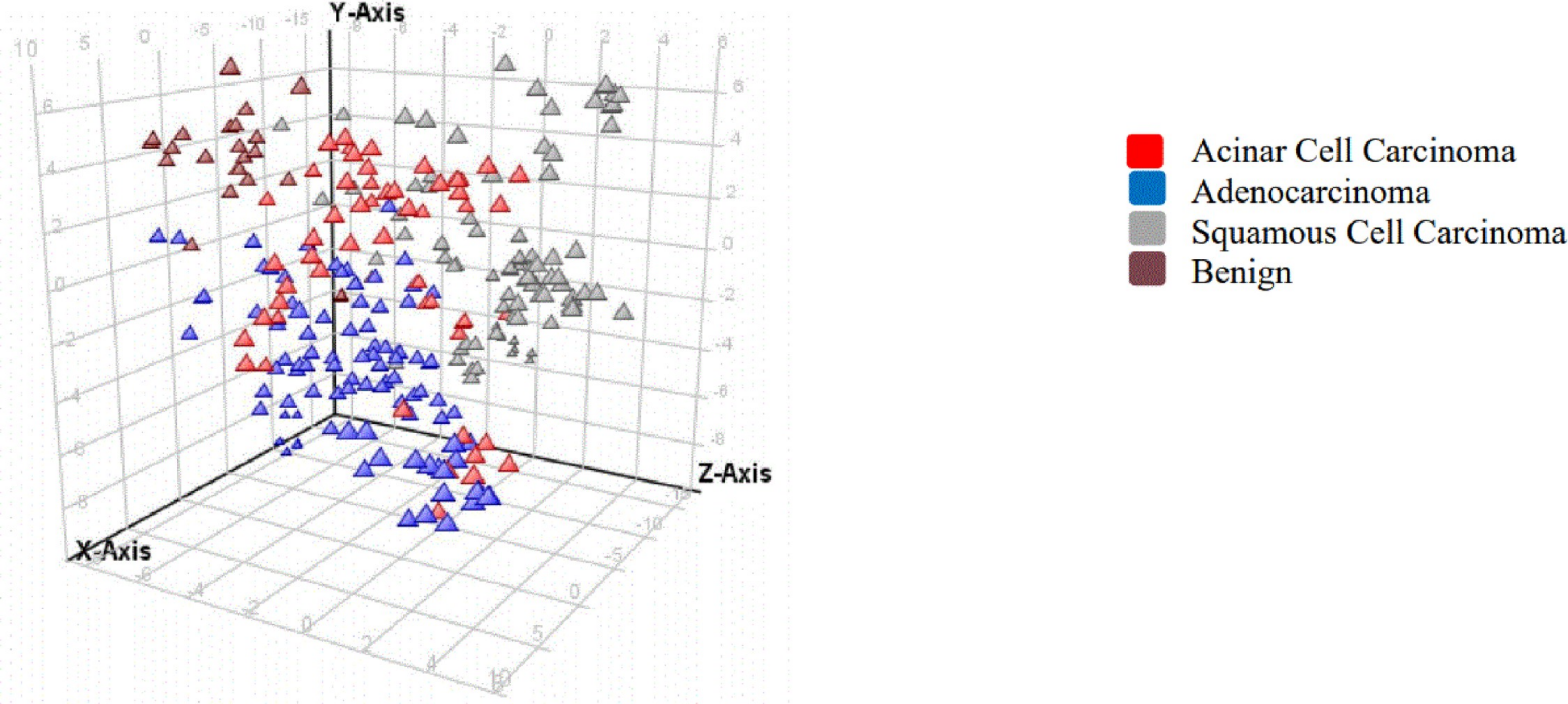
An overview of the t-scores from partial least squares discrimination (PLSD) analyses of plasma metabolites in cancer patients in ESI negative. Each triangle represents an individual patient (triplicate extraction and analysis).

**Fig 5:**
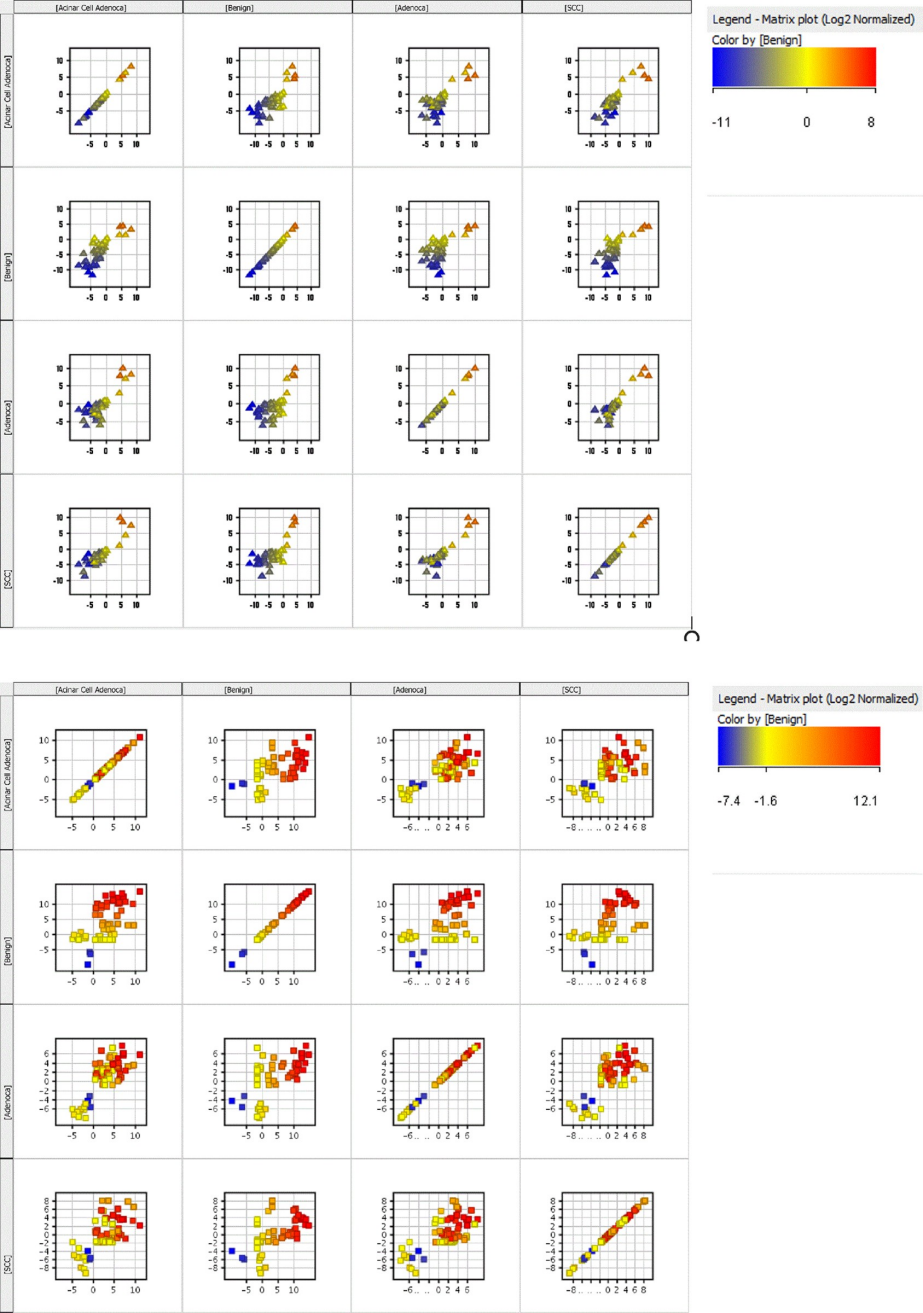
**a.** Matrix plot of pairwise 2D scatter plots for patients with benign and the three selected cancers (Esi Pos). **b.** Matrix plot of pairwise 2D scatter plots for patients with benign and the three selected cancers (Esi Neg).

**Step 1: Metabolomics analysis of blood sample extracts (LC-QTOF-MS and MassHunter Qualitative (MHQ B07)**
Non-targeted analysis of all extracts by LC-QTOF-MS in ESI Positive and Negative modes
Molecular Feature Extraction (MFE) algorithm was used to extract all detectable compounds
Find by Ion algorithm was used to remove false +/- compounds
1569 and 1789 entities were detected in ESI Positive and Negative, respectively
**Step 2: Search using the Metlin Database**
The Metlin Database was used to search all detected entities (matching to accurate mass and isotope ratios)
**Step 3: Statistical Analysis (MPP 12.6.1 and XLSTAT)**
**Three-way ANOVA** (p<0.05) followed by a multiple testing correction of p values (Bonferroni FWER) comparing all identified metabolites in plasma extracts after filtering by frequency (At least 50% of one of the 3 conditions to give 574 and 668 entities in ESI Positive and Negative, respectively) in 42 patients to examine the effect of collection tube (Streck vs Heparin), (Arterial vs Venous Blood) and (Female vs Male) **Results**: 96 metabolites were statistically significant (Fig 1_Heatmap; Fig 2_PCA Biplot; S1 Table)
**One-way ANOVA** (p<0.05) followed by a multiple testing correction of p values (Bonferroni FWER) comparing all identified metabolites in plasma extracts after filtering by frequency (At least 50% of one of the 4 conditions to give 968 and 848 entities in ESI Positive and Negative, respectively) in 42 patients divided into 4 groups based on the tumour pathology report (Benign (n = 3, All Female), Acinar Cell Adenocarcinoma (n = 7; 6 females;1 male), Squamous Cell Carcinoma (n = 11; 10 females;1 male) and Adenocarcinoma (n = 7; 6 females;1 male)) **Results**: 130 metabolites were statistically significant (Fig 3_Heatmap; Fig 4: PLSD; Fig 5A and 5B_Matrix Plots; S2 Table)

## Results and discussion

The characteristics of a cohort of 42 patients who underwent surgery for suspected lung cancer between July 2017 and June 2018 are shown in Table 1. Patient numbers were equally balanced, having 21 females, and 21 males in the cohort, ranging from 25 to 83 years of age. The Table includes information on cancer type based on the histology report, stage, and smoking status. The majority of patients in the cohort were diagnosed with AC (n = 22) or SCC (11), compared to the other types combined (n = 6) and benign tumours (n = 3). The total number of samples assayed was 168, with each sample analysed in triplicate.

Across the entire cohort a total of 1569 metabolites were identified in the ESI positive mode and 1787 metabolites in the ESI negative mode.

Comparisons were focused on determining significant differences between arterial and venous samples, differences between Streck and Heparin tubes, and differences due to sex. Data were filtered for further analysis to include only metabolites that were present in at least 50% of the samples in at least one of the three conditions (i.e. blood location, tube type and sex).

As summarized in S1 Table, three-way ANOVA revealed 96 metabolites that were affected by collection tube, blood sampling and sex, belonging to a wide array of compound classes. The majority of the detected compounds were affected by sex (n = 56), with blood location contributing to a minor difference (n = 8). A total of 33 compounds significantly affected by collection tube include: lipids (i.e., phospholipids, sphingolipid), acids (i.e., azoles, carboxylic, phenylpropanoic, and ketoacids), pharmaceutical agents (i.e., antibiotics, benzothiaphenes, benzenes), signalling molecules (i.e., eicosanoids), vitamins (i.e., D2, D3), steroids (i.e., oxocortisol), nucleotides, polyphenols, sugars (i.e., L-fucose), and other organic compounds (i.e., thienoimidazolidines, pyridines, purines, organosulfur, organooxygen, etc.). Out of the 33 compounds significantly affected by collection tube, 18 compounds were higher when the Streck tubes were used, and 15 were higher in the Heparin tubes. In general, larger metabolite intensities were observed in Streck tubes. Allantoin in Streck tubes (Log 2 values range 7000–8000) was the compound of highest disparity compared to the compound in Heparin tubes (Log 2 values range 1.2–2.2). Whereas, pyridinoline in Heparin tubes (Log 2 values range 18.7–116.8) was higher compared to the Streck tubes (Log 2 values range 0.5–0.8).

Streck tubes were developed for DNA stabilization and not for metabolites. Due to the unknown chemistry of the Streck tube materials due to its’ proprietary nature, we are limited in our ability to discuss the phenomena which we have observed in the current study.

A hierarchical heatmap applied to significant metabolites obtained from the three-way ANOVA studies is provided in Fig 1.

As shown in this clustering heatmap, the first layer of separation divided the 8 groups into 2 groups based on collection tubes used in this study which indicated that the BCT was the most critical condition to decipher these metabolites. Sex and blood location then divided each tube group into additional clusters. There are two noteworthy clusters exhibiting the same trend, as visible in the bottom half of the heatmap. The detected metabolites belonged to a variety of different classes, with phospholipids and carboxylic acids covering a total of 28% of all detected compounds (Table 3).

**Table 3:** Classification and frequency of 96 detected metabolites in blood samples collected from 42 patients based on their chemical taxonomy.

Chemical Taxonomy	Frequency (%)
Phospholipid	14.583
Carboxylic acid	13.542
Eicosanoid	6.250
Fatty acid metabolism	5.208
Drug (Benzene)	5.208
Sphingolipid	4.167
Organooxygen	3.125
Phenols	3.125
Polyphenol	2.083
Purine Nucleotide	2.083
Steroids	2.083
Sugar	2.083
Vitamin	2.083
Endocannabinoids	1.042
Azoles	1.042
Drug (Antibiotic)	1.042
Drug (Benzothiaphenes)	1.042
Flavonoids	1.042
Organosulfur	1.042
Pteridines	1.042
Pyridine	1.042
Pyridine nucleotides	1.042
Thienoimidazolidines	1.042
Thymine	1.042
Alcohol	1.042
Aldehyde	1.042
Alkaloids	1.042
Bile acid	1.042
Coenzyme	1.042
Creatine metabolism	1.042
Diazines	1.042
Disulfide	1.042
Dithianes	1.042
Drug (Carbazoles)	1.042
Fatty ester lipid	1.042
Glucuronides	1.042
Heme biproduct	1.042
Imidazopyrimidines	1.042
Mycotoxin	1.042
Organochlorine	1.042
Plant hormone	1.042
Pyrimidine Nucleosides	1.042
Sweetener	1.042
Tropane alkaloids	1.042
Tryptamine	1.042
Pheromone	1.042

The following compounds were more highly concentrated in Streck tubes compared to Heparin tubes: carboxylic acids (Met Cys, Tyr Met, nitrotyrosine), organooxygen compounds (urochloralic acid, erythritol), purine nucleotides (Oxo-dGMP), pyridine nucleotides (nicotinamide ribotide), dithianes (dimethyl-dithiane-diol), benzene (treprostinol) and dimethachlor (herbicide). The following compounds were higher in Heparin tubes: two isomers of PS 34:1, dopamine, and glucose.

The projection of the 8 active variables summarizing the study parameters (collection tube, blood collection, and sex) are presented in a PCA biplot (Fig 2).

These variables were correlated in two groups based on the main separating factor (BCT; Streck *vs*. Heparin) which expressed 90.48% of the variability represented by the first two axes F1 and F2. The two polarity modes used to ionize the extracted metabolites (ESI + and -) were then added to the PCA biplot to increase the interpretation quality showing a close range of the majority of the detected compounds in both polarity modes as evident by the proximity of the two ESI polarity centroids.

However, a few compounds gave different patterns. Allantoin and ketoleucine were detected at higher concentrations with Streck tubes while 3 metabolites; LysoPC(P-16:0), PS 40:6, and chenodeoxycholic acid glycine conjugate were mainly detected when collected using Heparin tubes.

Dependant on the clinical significance of the compounds involved, collection tubes must be chosen carefully. For instance, Streck tubes may be a better choice in blood collection due to the majority of better detection/ionization of these compounds. For instance, sugars such as L-fucose (6-deoxy-l-galactose) were found in higher concentrations in Streck tubes. As a free sugar in mammals, L-fucose is found in very low concentrations, but may serve as a marker in certain cancers, including lung cancer [4]. A MALDI-MS comparison of the sera from former and current smokers with non-small cell lung carcinoma (NSCLC) revealed that fucosylated glycans were increased in current smokers [4].

In a previous pilot study using a GC/MS metabolomics approach, ketoleucine was one of numerous metabolites that were down regulated in the serum of lung cancer patients as compared to healthy subjects [5]. Since this metabolite may serve as an indicator of cancer presence, it may be important to use Streck tubes in observing such changes, since the concentrations were much higher in these collection tubes in the current study. Another carboxylic acid, benzoic acid, was higher in Streck tubes. In a recent study, levels of benzoic acid were significantly changed (1.39 fold) in sera from lung cancer patients, in comparison to those in healthy volunteers [6]. In addition, benzoic acid was clustered with advanced stage lung cancer as seen in PLS-DA plots, suggesting that this metabolite increased with disease progression through metastasis or increased inflammation.

However, if lipids and vitamins are of interest, Heparin tubes should be selected. Phospholipid metabolic pathways are typically upregulated in lung cancer, yielding distinct signatures. Lysophosphatidylcholines (LysoPCs) are membrane lipids commonly upregulated in lung cancer patients, and they also exhibit proinflammatory properties [7]. In the current study, various phospholipids showed the most number of changes. Three LysoPCs were significantly changed based on our analysis, of which one, LysoPC(P-18:1) was different based on the collection tube (higher in Heparin tubes).

Multiple cellular signalling pathways mediate the effects of leukotriene B4, an eicosanoid, and one of the most potent leukotrienes, on the regulation of epithelial tumour cell proliferation, survival, migration, and invasion. Production of leukotriene B4 (LTB_4_), typically restricted to leukocytes, is synthesized in various diseased epithelial cells, including lung cancer cells [8, 9]. In a previous study, LTB4 concentrations in the serum of smokers were nearly 60-fold greater than those in nonsmokers [10]. However, lung cancer does present in cases of complete abstinence from smoking, so the link between serum LTB4, lung cancer and smoking must be carefully analysed. In the current study, carboxy-leukotriene B4, was notably higher in Heparin tubes.

Vitamin D may prolong cancer survival by inhibiting tumour progression and metastasis, however, there are limited studies regarding the association between circulating vitamin D metabolites and lung cancer survival. In the current study, three vitamin D metabolites hydroxyvitamin D3, dihydroxyvitamin D and vitamin D2 glucuronide) were higher in Heparin tubes. But since the majority of research has focused on 25-hydroxyvitamin D 25(OH)D, information on these metabolites is sparse. However, one study found that serum 25(OH)D was not associated with overall lung cancer survival in males [11].

Combining data from both collection tubes, and after filtering, one-way ANOVA of lung cancer types (Benign, AC, ACA, SCC) revealed a total of 130 significant (p<0.05) metabolites in both ESI positive and ESI negative modes combined (S2 Table). Classes of compounds of significance include: lipids (phospholipids, fatty acyls, glycerolipids), steroids, vitamins, organooxygen compounds, carboxylic acids, phenols, thiols, among others. Compounds showing the highest fold changes were from ESI positive mode. These results showed hydroxybenzoic acid significantly different between cancer types. In fact, its abundance in ACA was 2.4 times higher compared to benign tumours, and 5.2 times higher compared to AC. LysoPC(20:0) and LysoPC (22:4) showed the highest fold changes of 12.1 and 11.3 between benign and AC, respectively. LysoPCs 18:3), 18:3 and 20:1 were also significant. dihydroxyvitamin D had a 5.9 fold change between benign and AC, serving as a potential biomarker. Lactate is frequently observed as an upregulated metabolite in lung tissue and serum from lung cancer patients, as a higher glucose uptake and subsequent conversion to lactate is common in many tumours [12–17]. In a recent metabolomics study, lactate was observed to be up-regulated in SCC compared to AC [18]. We observed a similar effect, with lactic acid having 5.3 fold change. This effect is visible in the heatmap in Fig 3.

Fig 3 depicts a hierarchical heatmap generated for Log 2 Fold Change (FC) values for three types of cancers versus patients with benign tumours. Based on the metabolic profile provided here, patients with AC and ACA had a more similar metabolic profile. Individual metabolites with high abundance (red) that can be used as potential markers uniquely representing each of the three types of cancer used in our model may be used to either detect these types of cancers at early stages and/or employed as a tool to monitor the progress of these patients with potentially different treatments. SCC is showing a higher concentration of lactic acid compared to both ACA and AC. Since this has also been confirmed in literature, lactic acid is likely a suitable metabolic candidate or biomarker in differentiating between SCC and the other lung cancer types [18]. Very few other metabolites were also highly concentrated in SCC, such as dehydroxyzyleuton, glutarylcarnitine, 2-butanol and citrulline. ACA and AC, on the other hand, displayed a number of potential candidates for differentiation. These included LysoPC (P-16:0) in ACA, and LysoPC(18:3) in AC. As previously mentioned, the LysoPCs have been noted in literature to be elevated in the plasma of lung cancer patients [19].

A partial least square discrimination (PLSD) model was also used to decipher the 3 cancer groups from benign patients based on 130 significant metabolites (Fig 4).

This PLSD model had a prediction accuracy of 81%. Additional cancer patients belonging to each of the cancer type used in our study can provide further validation to this model. Although the use of Bonferroni FWER certainly reduces false positives, this model used for this pilot study may be further adjusted and validated in a larger sample set with additional benign and cancer patients.

A matrix plot was used to provide an overview of the correlation between different types of cancer. Matrix of pairwise 2D scatter plots for patients with benign and the three selected types of cancer is presented in Fig 5A and 5B for significant metabolites in the ESI positive and ESI negative modes, respectively.

These graphs further support the utility of these 130 significant compounds as a potential screening tool which can help with differentiation of the selected cancers from patients with benign tumours and/or from each other. This list can potentially be used in early diagnosis and/or continuous monitoring of the diagnosed patients for early detection of any potential recurrence.

The goal of metabolomics research in this area is to aid in early detection, and to identify potential biomarkers that can help distinguish between lung cancer and healthy patients, and various lung cancer types. Certain metabolites discussed in the current study may serve this function, and replicated studies are needed to confirm these metabolites as biomarkers. In addition, our subsequent experiment demonstrated that BCT had a major effect on the presence and concentration of metabolites, understandably since collection tube additives are important sources of pre-analytical errors [20]. Streck tubes contain K3EDTA as an anticoagulant while sodium Heparin is the anticoagulant in the Heparin vacutainers. More work is clearly needed to ensure such additives do not interfere with clinical assays. Based on these findings, it is important to use multiple tubes if a non-targeted metabolomics approach is selected. If a targeted approach is to be used, it is imperative that pre-testing of blood be used to ensure the most appropriate blood collection tubes are chosen for such assays, to ensure important biomarkers are not overlooked.

It is estimated that in 2020, 21,200 Canadians will die from lung cancer, and 29,800 will be newly diagnosed, making lung cancer one of the most prevalent types of cancer [1]. The small size of tumours in the early stages of cancer, and the absence of cellular specificity of almost all metabolites, makes detection of cancer-specific metabolites in early stages difficult. However, the power of metabolomics may allow for the non-invasive detection of early stages of lung cancer, and the current work may provide further insight into the preferred BCT that should be used.

## Supporting information

S1 Table Three-way ANOVA of compounds significantly affected by blood collection tube (Streck (S) *vs*. Heparin (H)), blood sampling (venous (V) *vs*. arterial (A), and sex (M *vs*. F). Each row represents a metabolite (total of 130 significant metabolites after statistical analysis; three-Way ANOVA; p<0.05; Bonferroni FWER). Values for each cell represents Log 2-normalized values for each metabolite. ESI polarity modes: Positive (+ve); Negative (-ve).
(DOCX)

S2 Table One-way ANOVA of compounds significantly affected by lung cancer type (Benign vs. Acinar Cell Adenocarcinoma (ACA), Adenocarcinoma (AC), and Squamous Cell Carcinoma (SCC)). Each row represents a metabolite (total of 96 significant metabolites after statistical analysis; One-Way ANOVA; p<0.05; Bonferroni FWER). Values for each cell represents Log 2 Fold Change (FC)-normalized values for the ratio of each of the three cancer types (SCC, ACA or AC)/ Benign. ESI polarity modes: Positive (+ve); Negative (-ve).
(DOCX)
