# Supplementary material for: Comparative metabolomics studies of blood collected in streck and heparin tubes from lung cancer patients
Source: PLoS One. 2021 Apr 23;16(4):e0249648. doi: 10.1371/journal.pone.0249648 (PMC8064553; doi:10.1371/journal.pone.0249648)
Supplement: S1 Table — Each row represents a metabolite (total of 130 significant metabolites after statistical analysis; three-Way ANOVA; p<0.05; Bonferroni FWER). Values for each cell represents Log 2-normalized values for each metabolite. ESI polarity modes: Positive (+ve); Negative (-ve). (DOCX) [file pone.0249648.s001.docx]

S1 Table. Three-way ANOVA of compounds significantly affected by blood collection tube (**Streck (S) *vs.* Heparin (H)**), blood sampling (**Venous (V) *vs.* Arterial (A**), and **Sex (M *vs.* F)**.

| **Compound** | **Polarity Mode** | **Formula** | **m/z** | **B** | **T** | **S** | **H, A, F** | **H, A, M** | **H, V, F** | **H, V, M** | **S, A, F** | **S, A, M** | **S, V, F** | **S,V, M** |
| --- | --- | --- | --- | --- | --- | --- | --- | --- | --- | --- | --- | --- | --- | --- |
| Cyclic GMP | -ve | C10H12N5O7P | 344.0425 |  | T |  | 0.8 | 0.6 | 0.5 | 0.8 | 6.7 | 3.7 | 10.8 | 1.6 |
| Hydroxychlorpropamide | -ve | C10H13ClN2O4S | 291.0301 |  | T |  | 0.8 | 0.6 | 0.5 | 0.6 | 1.7 | 1.3 | 5.3 | 1.9 |
| Hydroxyzileuton | -ve | C11H12N2O3S | 251.048 |  | T |  | 1.0 | 1.8 | 1.5 | 1.2 | 19.2 | 40.2 | 9.9 | 38.1 |
| Nicotinamide ribotide | -ve | C11H15N2O8P | 333.0363 |  | T |  | 2.2 | 0.7 | 0.9 | 0.6 | 5.5 | 10.7 | 6.5 | 7.1 |
| Benzoyl glucuronide | -ve | C13H14O8 | 297.0579 |  | T |  | 1.0 | 0.9 | 0.8 | 0.8 | 2118.8 | 1519.2 | 591.1 | 3985.6 |
| Pyridinoline | -ve | C18H28N4O8 | 427.1779 |  | T |  | 35.8 | 46.1 | 18.7 | 116.8 | 0.8 | 0.6 | 0.5 | 0.5 |
| Methylene-THF | -ve | C20H23N7O6 | 516.183 |  | T |  | 5.2 | 2.7 | 2.2 | 7.8 | 0.8 | 0.6 | 0.5 | 0.5 |
| Carboxy-leukotriene B4 | -ve | C20H30O6 | 411.1903 |  | T |  | 30.9 | 27.7 | 10.5 | 80.0 | 1.3 | 1.0 | 1.0 | 0.8 |
| Oxocortisol | -ve | C21H28O6 | 421.1991 |  | T |  | 454.4 | 291.8 | 49.4 | 152.9 | 9.1 | 11.4 | 5.9 | 44.9 |
| Oxytetracycline | -ve | C22H24N2O9 | 459.1351 |  | T |  | 383.4 | 25.7 | 32.4 | 6.3 | 4.4 | 0.9 | 0.8 | 3.0 |
| Malvidin glucoside | -ve | C23H25O12 | 538.1356 |  | T |  | 4.4 | 22.9 | 3.2 | 19.7 | 2.0 | 1.0 | 0.5 | 1.3 |
| Di-O-caffeoylquinic acid | -ve | C25H24O12 | 561.1128 |  | T |  | 13.0 | 2.7 | 2.9 | 3.0 | 0.8 | 0.6 | 0.5 | 0.6 |
| LysoPC(P-18:1) | -ve | C26H52NO6P | 504.3443 |  | T |  | 83.0 | 23.2 | 84.2 | 36.9 | 12.1 | 2.2 | 6.2 | 4.0 |
| Dihydroxyvitamin D | -ve | C27H44O3 | 415.3213 |  | T |  | 22.0 | 3.4 | 4.7 | 2.6 | 2.1 | 1.0 | 1.0 | 1.0 |
| Dimethylsulfide | -ve | C2H6S | 121.0298 |  | T |  | 1.2 | 0.8 | 0.6 | 1.2 | 3473.7 | 4635.0 | 986.2 | 6792.7 |
| Vitamin D2 -glucuronide | -ve | C34H52O7 | 571.3668 |  | T |  | 168.2 | 7.7 | 46.0 | 3.4 | 3.6 | 2.7 | 1.6 | 2.0 |
| Cer(d36:2) | -ve | C36H69NO3 | 608.5153 |  | T |  | 104.9 | 8.2 | 16.1 | 37.2 | 4.3 | 7.6 | 1.3 | 3.1 |
| PA(34:2) | -ve | C37H69O8P | 717.4716 |  | T |  | 11.1 | 4.3 | 1.8 | 8.4 | 1.0 | 0.7 | 0.8 | 0.8 |
| PA(34:1) | -ve | C37H71O8P | 673.4811 |  | T |  | 10.1 | 2.7 | 2.2 | 2.1 | 1.0 | 0.7 | 0.6 | 1.0 |
| Dihydroxyacetone phosphate | -ve | C3H7O6P | 168.9874 |  | T |  | 63.0 | 13.2 | 109.2 | 6.7 | 2471.3 | 1018.9 | 879.9 | 460.9 |
| PS(34:1) | -ve | C40H76NO10P | 760.5346 |  | T |  | 11.1 | 9.1 | 6.8 | 9.6 | 0.8 | 1.0 | 0.6 | 0.7 |
| PS(36:2) | -ve | C40H76NO10P | 760.5346 |  | T |  | 1.0 | 1.2 | 6.8 | 8.0 | 20.4 | 11.1 | 20.1 | 63.2 |
| PI(32:1) | -ve | C43H81O13P | 835.5645 | B | T |  | 2.7 | 2.6 | 2802.4 | 17363.1 | 3554.1 | 1606.0 | 1235.5 | 4917.7 |
| PS(34:1) | -ve | C43H81O13P | 835.5645 |  | T |  | 11.1 | 7.4 | 5.3 | 9.6 | 0.8 | 0.7 | 0.6 | 0.7 |
| Allantoin | -ve | C4H6N4O3 | 157.0367 |  | T |  | 2.2 | 1.8 | 1.5 | 1.2 | 23207.3 | 70626.8 | 7998.6 | 82630.1 |
| Pyroglutamic acid | -ve | C5H7NO3 | 128.0341 |  | T |  | 16.4 | 1.8 | 12.2 | 4.5 | 84.3 | 19.4 | 167.8 | 29.5 |
| Dihydrothymine | -ve | C5H8N2O2 | 127.0515 |  | T |  | 0.8 | 0.6 | 0.5 | 0.8 | 6.1 | 4.7 | 7.6 | 1.8 |
| Ketoleucine | -ve | C6H10O3 | 129.0553 |  | T |  | 1637.7 | 6058.2 | 1027.6 | 8514.2 | 24936.4 | 10544.4 | 16256.6 | 17270.1 |
| Fucose | -ve | C6H12O5 | 163.057 |  | T |  | 0.8 | 0.6 | 0.5 | 0.6 | 8.1 | 2.8 | 9.1 | 1.5 |
| Quinolinic acid | -ve | C7H5NO4 | 166.0159 |  | T |  | 11.9 | 16.4 | 16.8 | 20.4 | 679.4 | 303.9 | 257.2 | 552.2 |
| Dihydroxyphenylglycol | -ve | C8H10O4 | 169.0516 |  | T |  | 0.8 | 0.8 | 0.6 | 1.0 | 16.8 | 69.0 | 7.5 | 17.1 |
| Homovanillic acid | -ve | C9H10O4 | 181.0489 |  | T |  | 0.8 | 0.6 | 0.5 | 0.6 | 19.5 | 22.7 | 7.4 | 26.4 |
| Methyl bisnorbiotinyl ketone | -ve | C9H14N2O2S | 213.0691 |  | T |  | 0.8 | 0.6 | 0.5 | 0.6 | 1820.6 | 2036.5 | 406.8 | 1632.2 |
| Thymidine | -ve | C10H14N2O5 | 241.0872 |  |  | S | 5.2 | 0.9 | 3.8 | 0.7 | 5.6 | 0.9 | 3.6 | 0.8 |
| Ecgonine methyl ester | -ve | C10H17NO3 | 258.1285 |  |  | S | 123.5 | 4.1 | 68.3 | 2.8 | 33.5 | 3.5 | 62.5 | 6.0 |
| Methoxydimethyltryptamine | -ve | C13H18N2O | 263.1414 |  |  | S | 44.8 | 1.3 | 17.3 | 2.4 | 39.2 | 1.9 | 18.3 | 2.0 |
| Lidocaine | -ve | C14H22N2O | 233.1588 |  |  | S | 1.4 | 5.4 | 1.6 | 63.6 | 1.9 | 15.2 | 0.7 | 5.7 |
| Leukotriene B5 | -ve | C20H30O4 | 333.2072 |  |  | S | 1164.4 | 21.6 | 398.1 | 26.1 | 349.4 | 17.2 | 316.5 | 11.8 |
| Prostaglandin J2 | -ve | C20H30O4 | 333.2072 |  |  | S | 1487.8 | 21.6 | 686.2 | 26.1 | 349.4 | 17.2 | 316.5 | 11.8 |
| Dihydro-keto-PGE2 | -ve | C20H32O5 | 351.2122 |  |  | S | 111.4 | 9.9 | 41.6 | 8.8 | 60.9 | 3.5 | 55.4 | 7.6 |
| Prostaglandin D2 | -ve | C20H32O5 | 351.2122 |  |  | S | 142.0 | 9.9 | 41.6 | 8.8 | 60.9 | 3.5 | 55.4 | 7.6 |
| LysoPC(P-16:0) | -ve | C24H50NO6P | 478.3475 |  |  | S | 4187.5 | 179.0 | 1077.7 | 120.8 | 273.0 | 16.0 | 340.6 | 97.8 |
| Androstanediol glucuronide | -ve | C25H40O8 | 467.2637 |  |  | S | 23.3 | 3.0 | 26.0 | 3.6 | 27.2 | 2.2 | 17.0 | 2.4 |
| Androstanediol glucuronide | -ve | C25H40O8 | 467.2637 |  |  | S | 23.3 | 3.7 | 33.5 | 3.6 | 27.2 | 2.2 | 17.0 | 2.4 |
| Chenodeoxycholic acid glycine conjugate | -ve | C26H43NO5 | 448.3059 |  |  | S | 2831.3 | 21099.7 | 622.2 | 22907.2 | 2850.9 | 12909.7 | 1032.9 | 14380.4 |
| Pregnanediol-glucuronide | -ve | C27H44O8 | 495.2964 |  |  | S | 3.6 | 0.6 | 2.6 | 0.9 | 3.7 | 0.7 | 7.4 | 0.6 |
| PS(40:6) | -ve | C46H78NO10P | 834.5621 | B |  |  | 88.4 | 103.0 | 11430.7 | 87387.9 | 18812.0 | 32325.2 | 6219.1 | 37977.2 |
| Selenomethionine | -ve | C5H11NO2Se | 241.9534 |  |  | S | 23.7 | 1.1 | 12.1 | 1.3 | 67.8 | 3.5 | 32.4 | 4.3 |
| Oxo-dGMP | +ve | C10H14N5O8P | 364.0591 |  |  | S | 0.3 | -0.3 | 0.0 | -0.1 | 12.8 | 15.3 | 12.5 | 15.3 |
| Metanephrine | +ve | C10H15NO3 | 198.1095 |  |  | S | -7.7 | -2.2 | -6.6 | -2.5 | -6.6 | -4.0 | -7.4 | -4.6 |
| Indoleacetic Acid | +ve | C10H9NO2 | 176.0656 |  |  | S | 2.6 | 7.5 | 2.2 | 8.4 | 1.0 | 1.2 | -0.3 | 2.0 |
| N-Ribosylhistidine | +ve | C11H17N3O6 | 288.1114 |  |  | S | 6.7 | 0.7 | 4.3 | 2.6 | 5.5 | 0.7 | 3.6 | 1.1 |
| Ile Pro | +ve | C11H20N2O3 | 229.1529 |  |  | S | 1.3 | 4.1 | 1.6 | 5.3 | 2.3 | 5.4 | 2.4 | 5.5 |
| Difluoro-dodecadien-ol | +ve | C12H20F2O | 219.1476 |  |  | S | 3.6 | 0.8 | 1.2 | 0.6 | 6.5 | 4.3 | 4.8 | 5.5 |
| Dimethachlor | +ve | C13H18ClNO2 | 256.11 |  |  | S | 0.7 | 0.5 | 1.6 | 0.9 | 6.9 | 8.4 | 5.4 | 9.5 |
| Sulfolithocholylglycine | +ve | C14H20N2O4S | 157.0603 |  |  | S | 4.6 | 8.4 | 4.6 | 9.3 | 5.4 | 8.0 | 4.7 | 8.7 |
| Tyr Met | +ve | C14H20N2O4S | 157.0603 |  |  | S | 0.3 | 1.2 | 0.0 | 0.1 | 9.0 | 11.3 | 6.0 | 11.7 |
| Farnesol | +ve | C15H26O | 223.204 |  |  | S | 4.0 | 6.8 | 4.6 | 8.0 | 3.8 | 7.3 | 4.2 | 7.4 |
| Sphinganine 1-phosphate | +ve | C18H40NO5P | 382.2722 | B |  |  | 4.6 | 4.5 | 17.8 | 16.4 | 7.6 | 6.4 | 8.6 | 7.9 |
| Escitalopram | +ve | C20H21FN2O | 325.17 |  |  | S | 2.5 | 0.8 | 4.1 | 0.6 | 2.9 | 0.4 | 2.6 | 0.4 |
| Eicosatrienoic acid | +ve | C20H32O2 | 305.2463 |  |  | S | 8.4 | 8.9 | 7.5 | 9.5 | 4.1 | 5.4 | 5.2 | 6.8 |
| Arachidonic acid | +ve | C20H32O2 | 305.2463 |  |  | S | -5.3 | -2.9 | -4.2 | -0.8 | -7.1 | -6.1 | -8.6 | -3.2 |
| Heneicosanedioic acid | +ve | C21H40O4 | 357.2994 |  |  | S | 2.8 | 3.5 | 2.8 | 3.7 | 0.7 | 1.1 | -0.3 | 0.4 |
| HETE ethanolamide | +ve | C22H37NO3 | 364.2945 |  |  | S | 0.0 | 3.9 | 0.8 | 4.0 | 0.8 | 0.5 | 0.6 | 3.2 |
| O-Desmethylcarvedilol | +ve | C23H24N2O4 | 393.1813 |  |  | S | 3.6 | -0.3 | 2.7 | -0.1 | 3.3 | -0.3 | 2.4 | -0.3 |
| Treprostinil | +ve | C23H34O5 | 380.2545 |  |  | S | -10.6 | -9.4 | -9.5 | -6.2 | -5.8 | -1.8 | -3.6 | -2.6 |
| Arachidonylglycerol | +ve | C23H38O4 | 379.2783 | B |  |  | -8.6 | -7.0 | -0.5 | -1.4 | -7.0 | -8.5 | -4.5 | -4.0 |
| MG(20:4) | +ve | C23H38O4 | 396.3108 | B |  |  | -8.6 | -7.0 | -0.5 | -1.4 | -7.0 | -8.5 | -4.5 | -4.0 |
| LysoPC(22:4) | +ve | C30H54NO7P | 572.3692 |  |  | S | -6.8 | -2.4 | -8.0 | -2.2 | -8.6 | -7.1 | -10.4 | -5.7 |
| Bilirubin | +ve | C33H36N4O6 | 585.2696 |  |  | S | -2.7 | -0.5 | -1.5 | -0.3 | -7.8 | -4.8 | -7.6 | -3.9 |
| Janthitrem C | +ve | C37H47NO4 | 548.3635 |  |  | S | -2.2 | -1.2 | -0.6 | -1.5 | -5.6 | -4.3 | -7.1 | -4.8 |
| C16 Sulfatide | +ve | C40H77NO11S | 764.5353 |  |  | S | -9.5 | -13.0 | -4.5 | -5.7 | -2.8 | -5.0 | -5.9 | -1.2 |
| SM(d36:2 ) | +ve | C41H81N2O6P | 729.5898 | B |  |  | 47.7 | 14.1 | 736.6 | 3142.7 | 421.0 | 66.2 | 387.3 | 1781.2 |
| PS(36:3) | +ve | C42H76N)10P | 786.5197 |  |  | S | 12.3 | 13.7 | 10.7 | 6.8 | 5.5 | 6.1 | 6.4 | 4.4 |
| PE(41:6) | +ve | C46H80NO8P | 806.5674 |  |  | S | -10.1 | -10.6 | -1.1 | -2.1 | -2.3 | -1.8 | -1.8 | -2.7 |
| PC(o-38::6) | +ve | C46H82NO7P | 792.5861 | B |  |  | 5.1 | 3.0 | 9.9 | 10.7 | 7.3 | 6.6 | 8.7 | 10.4 |
| Erythritol | +ve | C4H10O4 | 123.065 |  |  | S | -8.1 | -7.2 | -7.4 | -7.5 | -1.4 | 0.5 | -2.1 | -0.7 |
| Cystamine | +ve | C4H12N2S2 | 153.0499 |  |  | S | 7.9 | 10.1 | 7.1 | 11.6 | 4.3 | 6.5 | 4.0 | 8.0 |
| Creatinine | +ve | C4H7N3O | 114.0662 |  |  | S | -2.8 | -0.2 | -2.0 | -0.1 | -2.7 | -0.7 | -3.1 | -0.1 |
| Coenzyme Q9 | +ve | C54H82O4 | 795.6074 |  |  | S | 4.9 | 6.1 | 0.9 | 0.2 | 0.0 | -0.3 | -0.3 | 0.2 |
| Ribulose | +ve | C5H10O5 | 151.0562 |  |  | S | 7.8 | 5.0 | 8.0 | 7.0 | 3.4 | 2.9 | 4.6 | 2.4 |
| -Dimethyl-dithiane-diol | +ve | C6H12O2S2 | 165.0534 |  |  | S | 1.1 | 1.9 | 1.9 | 2.6 | 12.0 | 11.9 | 9.8 | 10.7 |
| Glucose | +ve | C6H12O6 | 181.0708 |  |  | S | -1.0 | -0.2 | -0.2 | 0.0 | -4.8 | -1.8 | -5.8 | -2.2 |
| Gly Pro | +ve | C7H12N2O3 | 173.0946 |  |  | S | 2.6 | -0.3 | 2.8 | 1.4 | 8.5 | 5.9 | 7.4 | 6.3 |
| Heptanal | +ve | C7H14O | 115.1087 |  |  | S | 0.7 | 4.0 | 1.3 | 4.5 | 1.0 | 4.7 | -0.2 | 3.1 |
| Homocitrulline | +ve | C7H15N3O3 | 190.1213 | B |  |  | 0.0 | 0.0 | 4.0 | 0.6 | 0.0 | -0.3 | 2.5 | 1.2 |
| Acetylamino-formylamino-methyluracil | +ve | C8H10N4O4 | 227.0789 |  |  | S | 3.3 | 8.1 | 4.7 | 8.4 | 2.0 | 2.6 | 0.3 | 3.5 |
| Urochloralic acid | +ve | C8H11Cl3O7 | 346.9323 |  |  | S | 1.5 | 2.0 | 3.4 | 1.6 | 12.7 | 12.8 | 11.9 | 13.9 |
| Dopamine | +ve | C8H11NO2 | 154.0831 |  |  | S | 4.4 | 4.9 | 4.8 | 8.2 | 2.4 | 2.6 | 1.7 | 2.2 |
| Met Cys | +ve | C8H16N2O3S2 | 253.0651 |  |  | S | 1.0 | -0.3 | 1.4 | 0.4 | 15.4 | 15.3 | 13.8 | 15.8 |
| Caprylic acid | +ve | C8H16O2 | 145.1218 |  |  | S | 2.4 | 5.1 | 2.8 | 8.1 | 2.7 | 4.7 | 1.6 | 5.4 |
| Nitrotyrosine | +ve | C9H10N2O5 | 227.075 |  |  | S | 0.0 | -0.3 | 0.0 | -0.1 | 5.4 | 8.3 | 4.0 | 8.3 |
| Decanoylcarnitine | +ve | C17H33NO4 | 316.2472 |  |  | S | -6.7 | -3.9 | -8.4 | -2.6 | -6.4 | -4.1 | -6.3 | -3.2 |
| Dimethylaminopurine | +ve | C7H9N5 | 181.1207 |  |  | S | 4.6 | 8.2 | 5.0 | 9.1 | 5.9 | 10.0 | 3.9 | 8.4 |
| Heptanone | +ve | C7H14O | 115.1087 |  |  | S | 4.1 | 7.8 | 5.1 | 8.3 | 4.7 | 8.5 | 3.6 | 6.9 |
| PE(P-16:0) | +ve | C21H44NO6P | 438.2967 |  |  | S | 3.8 | 5.6 | 3.4 | 7.8 | 2.1 | 4.3 | 1.1 | 4.3 |

- Each row represents a metabolite (total of 96 significant metabolites after statistical analysis; Three-Way ANOVA; p<0.05; Bonferroni FWER)
- Values for each cell represents Log 2-normalized values for each metabolite
- ESI polarity modes: Positive (+ve); Negative (-ve)
