# Supplementary material for: Comparative metabolomics studies of blood collected in streck and heparin tubes from lung cancer patients
Source: PLoS One. 2021 Apr 23;16(4):e0249648. doi: 10.1371/journal.pone.0249648 (PMC8064553; doi:10.1371/journal.pone.0249648)
Supplement: S2 Table — Each row represents a metabolite (total of 96 significant metabolites after statistical analysis; One-Way ANOVA; p<0.05; Bonferroni FWER). Values for each cell represents Log 2 Fold Change (FC)-normalized values for the ratio of each of the three cancer types (SCC, ACA or AC)/ Benign. ESI polarity modes: Positive (+ve); Negative (-ve). (DOCX) [file pone.0249648.s002.docx]

S2 Table. One-way ANOVA of compounds significantly affected by lung cancer type (Benign vs. Acinar Cell Adenocarcinoma **(ACA)**, Adenocarcinoma **(AC)**, and Squamous Cell Carcinoma **(SCC)**).

| **Compound** | **ESI**  **Polarity** | **Formula** | **m/z** | **Log FC**  **(ACA *vs.* Benign)** | **Log FC (AC *vs.* Benign)** | **Log FC**  **(SCC *vs.* Benign)** |
| --- | --- | --- | --- | --- | --- | --- |
| Tetradecanoylcarnitine | +ve | C21H41NO4 | 372.3133 | 5.4 | 10.7 | 13.5 |
| Stearoylcarnitine | +ve | C25H49NO4 | 428.3735 | 7.7 | 9.5 | 10.5 |
| Ribose phosphate | +ve | C5H11O8P | 231.0259 | -3.4 | -4.3 | -12.4 |
| Resolvin D1 | +ve | C22H32O5 | 377.2307 | 1.9 | 7.6 | 6.7 |
| Proline betaine | +ve | C7H13NO2 | 144.1012 | -1.6 | 4.5 | 7.6 |
| p-Cresol glucuronide | +ve | C13H16O7 | 285.0954 | -4.6 | -7.0 | -15.5 |
| N-Ribosylhistidine | +ve | C11H17N3O6 | 288.1114 | 3.0 | 2.2 | -4.4 |
| Myristic acid | +ve | C14H28O2 | 229.2152 | 1.5 | 7.9 | 8.8 |
| Metanephrine | +ve | C10H15NO3 | 198.1095 | 1.4 | 8.4 | 10.3 |
| LysoPC(22:4) | +ve | C30H54NO7P | 572.3692 | 4.3 | 11.3 | 11.2 |
| LysoPC(20:1) | +ve | C28H56NO7P | 550.3842 | 3.5 | 6.6 | 5.7 |
| LysoPC(20:0) | +ve | C28H58NO7P | 552.4014 | 5.3 | 12.1 | 9.9 |
| LysoPC(18:3) | +ve | C26H48NO7P | 518.3226 | 4.3 | 7.0 | 5.3 |
| Janthitrem C | +ve | C37H47NO4 | 548.3635 | 4.8 | 7.3 | 7.0 |
| Heptanal | +ve | C7H14O | 115.1087 | 3.2 | 9.7 | 13.0 |
| Glycoursodeoxycholic acid | +ve | C26H43NO5 | 450.3203 | 0.8 | 5.7 | -1.6 |
| Epinephrine glucuronide | +ve | C15H21NO9 | 360.1245 | 2.3 | 9.6 | 3.2 |
| Ecgonine methyl ester | +ve | C10H17NO3 | 200.1236 | -1.2 | 3.4 | 0.6 |
| Dodecanoylcarnitine | +ve | C19H37NO4 | 344.2782 | -0.1 | 6.3 | 9.7 |
| Docosahexaenoic acid | +ve | C22H32O2 | 329.2470 | 5.6 | 7.4 | 7.1 |
| Deoxycorticosterone | +ve | C21H30O3 | 331.2187 | 2.4 | 6.1 | 9.7 |
| Deoxycholic acid glycine conjugate | +ve | C26H43NO5 | 450.3203 | 0.8 | 5.8 | -1.6 |
| Dehydroxyzyleuton | +ve | C11H12N2OS | 221.0723 | 5.8 | 5.4 | 7.0 |
| Decanoylcarnitine | +ve | C17H33NO4 | 316.2472 | -0.2 | 7.0 | 5.3 |
| Cystamine | +ve | C4H12N2S2 | 153.0499 | 5.2 | 9.0 | 9.7 |
| Octenedioic acid | +ve | C8H12O4 | 173.0793 | 6.0 | 1.5 | -1.8 |
| Chenodeoxycholic acid glycine conjugate | +ve | C26H43NO5 | 450.3203 | 0.8 | 5.8 | -1.6 |
| Caprylic acid | +ve | C8H16O2 | 145.1218 | -0.6 | 6.1 | 3.5 |
| Capric acid | +ve | C10H20O2 | 173.1529 | 3.8 | 5.4 | -1.3 |
| Calcitriol | +ve | C27H44O3 | 417.3351 | 4.5 | 5.9 | 6.4 |
| Caffeine | +ve | C8H10N4O2 | 195.0875 | 9.9 | 8.2 | 5.6 |
| Butenylcarnitine | +ve | C11H19NO4 | 230.1403 | 5.7 | 2.5 | 4.3 |
| Betaine | +ve | C5H11NO2 | 118.0858 | 3.3 | 9.2 | 8.9 |
| Eicosatrienoic acid | +ve | C20H34O2 | 307.2599 | 3.9 | 9.2 | 8.6 |
| Dimethylaminopurine | +ve | C7H9N5 | 181.1207 | 1.4 | 11.5 | 11.5 |
| Hydroxyhexanoic acid | +ve | C6H12O3 | 133.0840 | 2.3 | 6.8 | 2.3 |
| Hydroxypropofol | +ve | C12H18O2 | 195.1356 | -0.3 | 5.8 | 3.7 |
| Heptanone | +ve | C7H14O | 115.1087 | 3.2 | 9.7 | 12.7 |
| Ethylphenol | +ve | C8H10O | 123.0824 | -0.3 | 2.8 | 3.7 |
| Methoxybenzenepropanoic acid | +ve | C10H12O3 | 181.0850 | -1.0 | 4.3 | -0.6 |
| Octenal | +ve | C8H14O | 127.1112 | 7.9 | 11.5 | 6.3 |
| Butanol | +ve | C4H10O | 97.0636 | 6.4 | 8.6 | 10.8 |
| Hydroxyprogesterone | +ve | C21H30O3 | 331.2187 | 2.1 | 6.0 | 9.7 |
| Trihydroxybenzene | +ve | C6H6O3 | 127.0376 | 7.6 | 10.7 | 10.6 |
| Propylpent-enoic acid | +ve | C8H14O2 | 143.1074 | 0.0 | 0.2 | 6.5 |
| Vitamin K1-epoxide | -ve | C31H46O3 | 465.3417 | 3.9 | 4.2 | -0.2 |
| Vitamin A | -ve | C20H30O | 571.4518 | 2.7 | 3.7 | -0.2 |
| Tyrosine | -ve | C9H11NO3 | 180.0644 | -1.4 | -4.1 | -12.1 |
| Tryptamine | -ve | C10H12N2 | 159.0868 | 4.1 | 3.9 | -0.2 |
| Hydroxycotinine glucuronide | -ve | C16H20N2O8 | 367.1073 | -4.7 | -6.5 | -13.0 |
| Thymidine | -ve | C10H14N2O5 | 241.0872 | 4.3 | 0.8 | 6.2 |
| Thromboxane B2 | -ve | C20H34O6 | 369.2259 | 2.1 | 3.7 | -1.5 |
| TG(48:2) | -ve | C51H94O6 | 801.6920 | -0.6 | -3.0 | -10.2 |
| Terbutaline | -ve | C12H19NO3 | 449.2632 | -2.5 | -5.5 | -6.6 |
| Taurine | -ve | C2H7NO3S | 124.0103 | 5.3 | 2.2 | -0.2 |
| Suberylglycine | -ve | C10H17NO5 | 230.1022 | 4.9 | 5.1 | 0.9 |
| Selenomethionine | -ve | C5H11NO2Se | 241.9534 | -4.8 | -10.3 | -15.3 |
| S-Adenosylmethionine | -ve | C15H23N6O5S | 398.1448 | 2.2 | 3.5 | 0.0 |
| Retinoic acid | -ve | C20H28O2 | 345.2048 | 2.5 | 4.3 | -0.2 |
| Retinal | -ve | C20H28O | 567.4208 | 3.1 | 3.5 | -0.2 |
| Pyroglutamic acid | -ve | C5H7NO3 | 128.0341 | -4.2 | -8.8 | -10.1 |
| Prostaglandin A2 | -ve | C20H30O4 | 333.2072 | -5.5 | -6.3 | -12.5 |
| Pregnenolone sulfate | -ve | C21H32O5S | 395.1899 | 0.5 | -1.3 | 5.7 |
| Pregnenolone | -ve | C21H32O2 | 631.4807 | -2.0 | -6.1 | -2.2 |
| para-Trifluoromethylphenol | -ve | C7H5F3O | 161.0208 | -5.2 | -2.6 | -10.2 |
| Norethynodrel | -ve | C20H26O2 | 297.1918 | 4.9 | 2.8 | 0.2 |
| Nicotinic acid | -ve | C6H5NO2 | 168.0275 | 2.3 | -3.4 | -3.7 |
| Nicotine-N-oxide | -ve | C10H14N2O | 355.2119 | 2.9 | 3.7 | -0.2 |
| Niacinamide | -ve | C6H6N2O | 121.0450 | 3.8 | 3.7 | -0.2 |
| N1-Acetylspermine | -ve | C12H28N4O | 303.2333 | -4.7 | -7.0 | -6.7 |
| Methionine sulfoxide | -ve | C5H11NO3S | 164.0354 | 4.2 | 1.0 | 7.7 |
| LysoPC(P-16:0) | -ve | C24H50NO6P | 478.3475 | 5.6 | 0.9 | 1.4 |
| Lithocholic acid glycine conjugate | -ve | C26H43NO4 | 432.3127 | -7.2 | -7.8 | -13.2 |
| Lactic acid | -ve | C3H6O3 | 89.0243 | 0.3 | -0.3 | 7.2 |
| Hypotaurine | -ve | C2H7NO2S | 154.0188 | -3.6 | -8.6 | -17.1 |
| Hydroxyvalproic acid | -ve | C8H16O3 | 379.2331 | 3.4 | 3.3 | -0.2 |
| Hydroxytestosterone | -ve | C19H28O3 | 349.2055 | -6.7 | -7.6 | -9.6 |
| Hydroxycholesterol | -ve | C27H46O2 | 803.6769 | 1.6 | -0.5 | -5.0 |
| Hydrogen carbonate | -ve | CHO3 | 180.9978 | 3.6 | 3.7 | -0.2 |
| Homoserine | -ve | C4H9NO3 | 164.0570 | 3.8 | 3.8 | -0.2 |
| Glycylproline | -ve | C7H12N2O3 | 171.0770 | 6.1 | 4.0 | -0.2 |
| Glycerol phosphate | -ve | C3H9O6P | 171.0121 | -0.9 | -6.8 | -10.0 |
| Glutarylcarnitine | -ve | C12H21NO6 | 595.2888 | -3.6 | -4.6 | -0.4 |
| Glucosamine | -ve | C6H13NO5 | 178.0745 | 3.7 | 4.1 | 0.5 |
| Gemfibrozil | -ve | C15H22O3 | 249.1492 | -1.5 | -5.3 | -6.2 |
| Gamma-Aminobutyric acid | -ve | C4H9NO2 | 265.1481 | 6.0 | 9.0 | 6.0 |
| Fructose bisphosphate | -ve | C6H14O12P2 | 339.0038 | 4.2 | 3.4 | -0.2 |
| FAD | -ve | C27H33N9O15P2 | 1615.3151 | 3.5 | 3.5 | -0.2 |
| Equol | -ve | C15H14O3 | 241.0872 | 4.3 | 0.8 | 6.5 |
| Epinephrine | -ve | C9H13NO3 | 411.1774 | 3.3 | 3.7 | -0.2 |
| Ecgonine methyl ester | -ve | C10H17NO3 | 258.1285 | -7.6 | -8.5 | -9.8 |
| Dihydrogenistein | -ve | C15H12O5 | 271.0660 | 3.7 | 2.6 | -0.2 |
| Dihydrofolic acid | -ve | C19H21N7O6 | 442.1522 | 3.8 | 4.4 | 0.0 |
| Prostaglandin J2 | -ve | C20H30O4 | 333.2072 | -5.5 | -6.7 | -12.5 |
| dCTP | -ve | C9H16N3013P3 | 465.9889 | -4.0 | -6.9 | -12.6 |
| Citrulline | -ve | C6H13N3O3 | 395.1898 | 1.9 | 2.1 | 7.3 |
| Citric acid | -ve | C6H8O7 | 251.0453 | -9.5 | -8.3 | -13.0 |
| Chloroform | -ve | CHCl3 | 162.9121 | 8.3 | 5.6 | 8.7 |
| Chenodeoxycholic acid sulfate | -ve | C24H40O7S | 471.2421 | -2.0 | -4.8 | -8.5 |
| Campestanol | -ve | C28H50O | 803.7716 | -6.2 | -7.3 | -13.0 |
| Calcidiol | -ve | C27H44O2 | 399.3266 | -8.0 | -3.0 | -5.7 |
| Azelaic acid | -ve | C9H16O4 | 187.0978 | 1.6 | 3.2 | -1.8 |
| Asparagine | -ve | C4H8N2O3 | 131.0458 | 6.3 | 0.5 | 5.1 |
| Androstanedione | -ve | C19H28O2 | 333.2070 | -3.4 | -6.0 | -6.7 |
| N-Phenylacetyl-fructoseglutamine | -ve | C13H16N2O4 | 263.1035 | 5.0 | 0.2 | 6.9 |
| Alanyltryptophan | -ve | C14H17N3O3 | 274.1219 | 3.2 | 2.8 | -0.2 |
| AFN911 | -ve | C29H33N7O2 | 510.2529 | 4.3 | 1.9 | -0.2 |
| Acetyl-N-formyl-methoxykynurenamine | -ve | C13H16N2O4 | 263.1035 | 4.8 | 0.2 | 6.9 |
| HODE | -ve | C18H32O3 | 295.2280 | -8.4 | -7.4 | -11.9 |
| Hydroxy-methylguanine | -ve | C6H17N5O2 | 180.0536 | -7.0 | -5.8 | -14.4 |
| Dehydrotestosterone glucuronide | -ve | C25H34O8 | 507.2233 | 4.8 | 5.7 | -0.4 |
| Hydroxyindoleacetic acid | -ve | C10H9NO3 | 190.0512 | -6.8 | -7.3 | -11.9 |
| Eicosatrienoic acid | -ve | C20H34O2 | 305.2491 | -4.7 | -6.4 | -6.3 |
| Hydroxypropofol | -ve | C12H18O2 | 193.1227 | -6.3 | -2.7 | -8.1 |
| Hydroxybenzoic acid | -ve | C7H6O3 | 137.0244 | 2.4 | -2.8 | -2.0 |
| Hydroxybenzaldehyde | -ve | C7H6O2 | 121.0297 | -10.2 | -10.4 | -13.0 |
| Methoxytyrosine | -ve | C10H13NO4 | 210.0861 | -1.2 | -4.5 | -10.2 |
| Methoxybenzenepropanoic acid | -ve | C10H12O3 | 225.0681 | 0.0 | 1.7 | -6.2 |
| Methoxy-Hydroxyphenylglycol sulfate | -ve | C9H12O7S | 263.0257 | -1.5 | -7.1 | -11.7 |
| Hydroxytetradecanedioic acid | -ve | C14H26O5 | 593.3676 | -0.9 | -6.8 | -7.3 |
| Hydroxypicolinic acid | -ve | C6H5NO3 | 138.0209 | -3.6 | -8.7 | -17.7 |
| Hydroxydodecanoic acid | -ve | C12H24O3 | 215.1673 | 3.4 | 2.9 | -0.2 |
| Hydroxy-octadecenoylcarnitine | -ve | C25H47NO5 | 486.3491 | 4.0 | 3.4 | -0.2 |
| Alpha-Androstanediol glucuronide | -ve | C25H40O8 | 467.2637 | -4.9 | -7.9 | -15.5 |
| Diiodothyronine | -ve | C15H13I2NO4 | 1108.8188 | 3.9 | 4.5 | -0.2 |
| Androstanediol glucuronide | -ve | C25H40O8 | 467.2637 | -4.9 | -7.9 | -15.3 |
| Trimethoxyphenyl propanoic acid | -ve | C12H16O5 | 239.0924 | 0.3 | 1.6 | -6.9 |
| Diaminosalicylic acid | -ve | C7H8N2O3 | 167.0469 | 3.8 | 4.1 | -0.2 |
| Hydroxyandrostane-glucuronide | -ve | C25H40O8 | 467.2637 | -4.9 | -7.9 | -15.5 |
| Estradiol-glucuronide | -ve | C24H32O8 | 447.1979 | 4.0 | 5.7 | -0.8 |

**Notes**:

- Each row represents a metabolite (total of 130 significant metabolites after statistical analysis; One-way ANOVA; p<0.05; Bonferroni FWER)
- Values for each cell represents Log 2 Fold Change (FC)-normalized values for the ratio of each of the three cancer types (SCC, ACA or AC)/ Benign
- ESI polarity modes: Positive (+ve); Negative (-ve)
